# Coastal dynamism in Southern Thailand: An application of the CoastSat toolkit

**DOI:** 10.1371/journal.pone.0272977

**Published:** 2022-08-24

**Authors:** Jerome Curoy, Raymond D. Ward, John Barlow, Cherith Moses, Kanchana Nakhapakorn

**Affiliations:** 1 Centre for Aquatic Environments, University of Brighton, Brighton, United Kingdom; 2 Department of Landscape Management, Estonian University of Life Sciences, Tartu, Estonia; 3 Department of Geography, University of Sussex, Brighton, United Kingdom; 4 Department of Geography and Geology, Edgehill University, Ormskirk, United Kingdom; 5 Faculty of Environment and Resource Studies, Mahidol University, Nakhon Pathom, Thailand; Đại Học Duy Tân: Dai Hoc Duy Tan, VIET NAM

## Abstract

In Thailand, 17% of the population lives by the coast, approximately 11 million people. A combination of coastal erosion, sea level rise and coastal land subsidence are critical issues threatening the livelihoods of coastal communities. Thailand has invested a lot of money and installed conservation policies to restore and protect coastal mangroves and realign or replenish their beaches. This study assessed the use of the toolkit Coastsat to digitise a time series of shoreline positions from open access satellite images between 1990 and 2019 along 560 km of coastline in the provinces of Krabi and Nakhon Si Thammarat (NST). Based on these digitised shorelines and the use of the software Digital Shoreline Analysis System (DSAS), it was possible to identify shoreline change, which varied between -66 to +16.4 m/y in the mangroves of NST and -22.2 to +10.6 m/year on its sandy beaches. Shoreline change rates along the Krabi coast varied -34.5 to +21.7 m/year in the mangroves and -4.1 to +4 m/year on sandy beaches. Analysis of the spatial and temporal variations of the shoreline position during the survey period reveals a linkage between extreme weather conditions and coastal erosion along the NST coast while that linkage is less clear along the Krabi coast. CoastSat delivers crucial and accurate time series shoreline data over extensive areas that are vital to coastal managers and researchers in a completely remote manner, which is key with the presence of COVID-19 travel bans.

## 1 Introduction

Coastal areas are often densely populated and especially so in Thailand, and these represent a tremendous resource for the country in regards to recreational, economical, agricultural, environmental and tourism activities. Within the context of climate change, increasing rates of sea level rise and storm surges, widespread erosion and increasing anthropogenic pressures on coastal systems [1–4] the study of shoreline positions is a key parameter to understand the historical evolution of coastal environments and the influence of coastal processes [5–7]. The demarcation of the shoreline is one of the most rapidly changing geomorphic features in coastal areas and short- or long-term assessments are essential to mitigate or even reverse coastal erosion [8] especially at large spatial scales.

Data-scarcity is however a challenge for a lot of coastal studies as long-term datasets at large spatial scales are rare [9–13]. In an attempt to overcome those limitations, scientists investigating shoreline changes have turned to the use of satellite images [14–17] and many have preferred the use of open access images such as LandSat or Sentinel despite a compromise in resolution when compared with commercial image products [12, 17–20]. More recently, the development of the Google platform Earth Engine and machine learning algorithms has allowed the rapid processing of large volumes of open access satellite data back to the 70s [18–20]. In the context of COVID 19 and travel bans across countries, the use of these tools has become extremely useful for international research.

Sea level change along the coast of Thailand has been of interest since the late 80s and a lot of attention was directed on coastal areas around the Gulf of Thailand [21–24]. Over the last 20000 years until the late 80s, it was estimated that sea level had risen by 120 m [25]. A range of researchers have reported that Thailand is suffering from problems associated with coastal erosion [26–30] and land subsidence [30, 31]; and sea level rise linked to climate change compounds the pressure on coastal areas. [24] investigated variations in sea level along the Gulf of Thailand between 1985 and 2009, indicating that net sea level rise in Southern Thailand was approximately 1.4 mm/year.

[32] estimated that between 1979 and 1996 Thailand lost more than 50% of its mangrove areas driven as a result of degradation, sea level rise, river damming and sediment starvation [33]. Mangroves provide a wide range of ecosystem services including supporting biodiversity, carbon storage and sequestration, locking away pollutants [34–38], and they also provide coastal protection [39], as was noted following the 2004 tsunami in south east Asia [40–42]. In the context of mangrove recession, the Thai government and local authorities have been working together to reverse that trend by passing strong conservation laws and restoration campaigns since 1996. Numerous studies have also highlighted the alarming rates of beach erosion in Thailand, measuring rates of retreat ~33.65 m/year along sections of the Nakhon Si Thammarat (NST) coast [29]. Since then, the government, local authorities, stakeholders and coastal experts have worked together to mitigate or reverse the trend.

The aim of this study is to provide information on shoreline position changes along the Krabi and the NST coastlines in Southern Thailand using open access satellite data, machine learning toolkits (CoastSat; [19]) and semi-automated software (Digital Shoreline Analysis System (DSAS); [43]). Based on the shoreline’s demarcations in time series between 1990 to 2019, this study will look at spatial and temporal variations in shoreline positions and investigate the link between extreme weather events and coastal erosion.

## 2 Regional setting and geology

**Fig 1:**
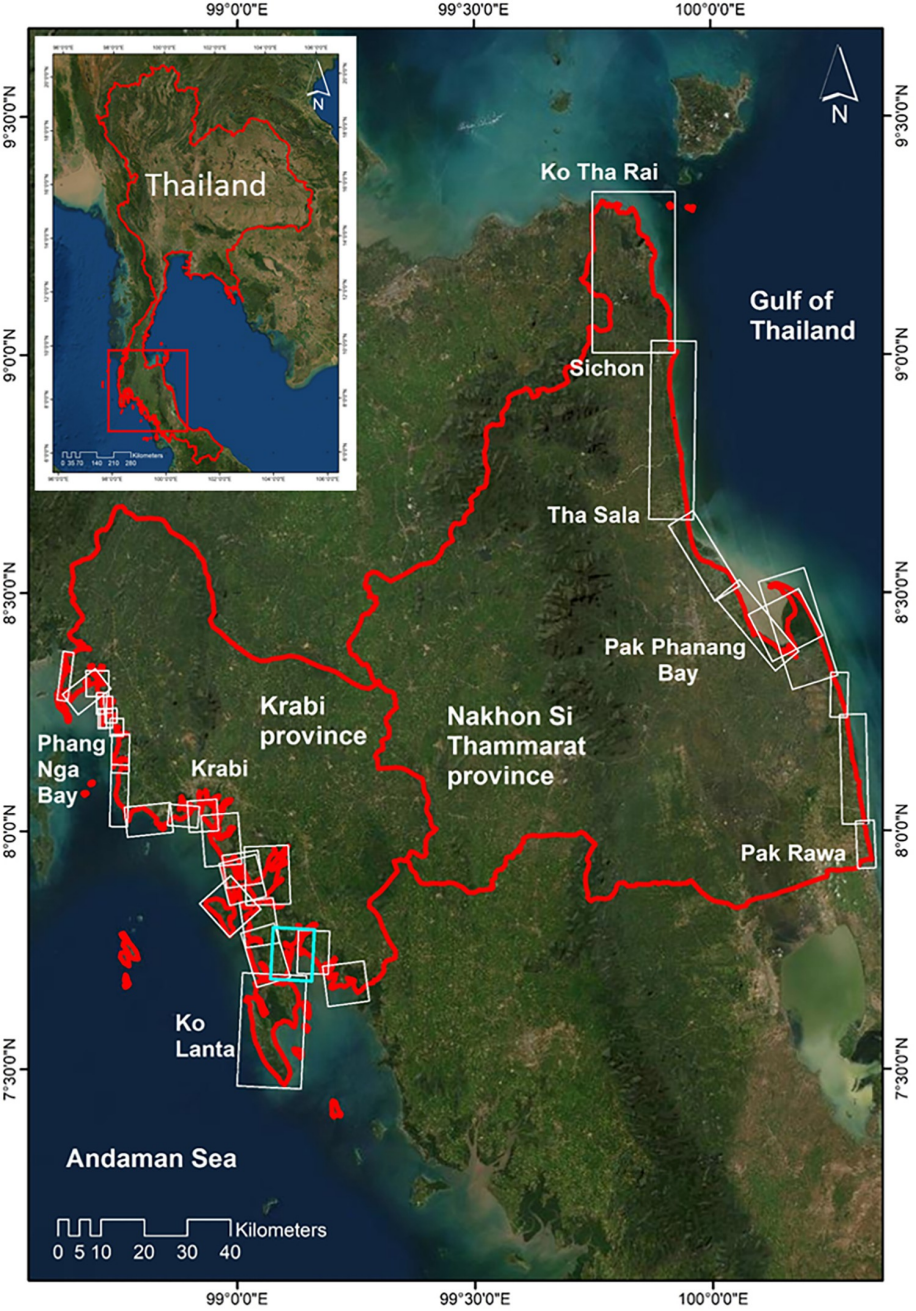
Location of the provinces of interest in Southern Thailand and identification of the coastal cell divisions for data processing along both coastlines. The blue contoured area is the only area where manual digitisation was necessary. Basemap satellite images accessed from World Imagery ESRI Tile Layer. Credits: Esri, Maxar, GeoEye, Earthstar Geographics, CNES/Airbus DS, USDA, USGS, AeroGRID, IGN, and the GIS User Community.

Southern Thailand is located between the Gulf of Thailand and the Andaman Sea. The coastline within the NST province extends 236.82 km and 203.79 km within the Krabi province (not including islands; [44]). The shape of the coastline in Southern Thailand is highly sinuous in the Krabi province whereas the coast in the Nakhon Si Thammarat province is noticeably less undulating (Fig 1). Both coastlines present a variety of environments: rocky cliffs; sand or gravel beaches; lagoons; wetlands; estuaries and tidal flats, although the Krabi coastline has a greater spatial variability. Estuarine and tidal flat areas are often colonised by mangroves and seagrasses, both valuable conservation areas for the South of Thailand. However, they also are important economically for tourism, recreational activities, fisheries/aquaculture industry; activities that also threaten their sustainability.

The climate in Southern Thailand is tropical with a regular occurrence of tropical storms, typhoons or hurricanes and two monsoons per year. The Southwest monsoon occurs from May to October bringing moderate to heavy rains and strong winds to the areas of interest. The retreat of the Southwest monsoon in September/October is frequently accompanied by extreme stormy weather, tropical cyclones or even hurricanes along the Nakhon Si Thammarat coast. They usually occur three to four times a year carrying heavy rain [45], causing flash flooding and enhancing coastal flooding. Strong winds from those events generate high energy waves travelling from the China Sea and breaking directly on the Southern coast of Thailand [46].

Coastal processes in Thailand are mainly controlled by waves and tides [21]. Significant wave heights in the Andaman Sea are generally <2m [46, 47]. That and the morphology of the Western coast of Thailand make wave conditions on the coast of the mainland relatively calm [48] with a significant wave height of approximately 0.74 m [46]. Along the Krabi coast, waves are most active during the Southwest monsoon from May to October with an average significant wave height of 1.17 m [46].

The influence of the Northeast monsoon is dominant on the East coast from November to March with an average significant wave height varying from 0.23 to 0.51 m depending on location along the NST coast. During calm conditions, significant wave heights vary from 0.11 to 0.24 m along the coastline [46]. Tides along both coasts of interest are mostly mixed semi-diurnal [49–52] and the tidal range on the West coast range from 1.1 to 3.6 m (micro- meso- tidal) whereas it ranges from 0.3 to 1.1m (micro-tidal) on the East coast [50].

Currents in the Southern part of the Gulf of Thailand are very complex resulting from the interaction of various parameters such as the tide, winds, monsoons, and currents from the China Sea. In general, tidal currents flow Northward during high tide and reverse as the tide changes. The strength of those currents varies with the tidal range, however tidal currents contribute very little to the net circulation within the Gulf [53]. In fact, the predominant monsoonal winds cause eddies, mixings and drive the exchange of the water mass in the Gulf [49, 53–55]. During the Northeast monsoon period, the strong Northeast winds build a strong flow to the Southwest at the Gulf entrance. When these currents encounter the coast, they deviate Northward following the coast. During the Southwest monsoon the strong West to Southwest winds from the Indian Ocean cause northward flows pushing water and currents outside of the Gulf. The circulation pattern during this period goes from North to South along the NST coast [55]. In the Andaman Sea, tidal currents are dominant in the Malacca Strait [56] and therefore along the Krabi coast. Surface currents in the Andaman Sea and the Malacca Strait are also influenced by seasonal monsoons. However, in the more local context of the Krabi coast, surface flow is predominantly directed North-westward towards the Andaman Sea, for both Southwest and Northeast monsoons [56, 57].

## 3 Methodology

Shoreline positions were extracted using the automated toolkit CoastSat [19]. CoastSat is an open-source software written in Python running on Anaconda 3 or other Python editor extracting free satellite data from Google Earth Engine and delineating shorelines on any coast in the world for a determined survey period. The toolkit digitises the boundary between sand and water at a sub-pixel level (10–15 m) based on the border segmentation of [58]. To further refine this technique, CoastSat introduces a new image classification, sharpening shoreline detection. As such, its algorithm operates on a sub-pixel resolution border segmentation and uses four classes of image classification. The precision of the digitised shorelines is increased by the ability of the user to manually draw the position of a “reference” shoreline on a cloudless image before the toolkit runs any automatic digitisation in order to avoid false detections [19]. During image classification, each pixel is classified in one of four categories (sand, white-water, water, and others such as vegetation). The pixel identification proved to be 99% accurate [14]. The boundary between sand and water is then extracted using a Modified Normalized Difference Water Index (MNDWI). Each digitised shoreline is visualised using 3 different backgrounds, RGB satellite image, output of image classification (sand, white-water, water, and others) and a grayscale image of the MNDWI pixel values. These three visual supports help the user in verifying and validating the accuracy of the digitisation for each analysed satellite image and the consistency of the pixel classification allows comparison between satellite images. This study uses satellite images acquired by Landsat 5, 7, 8 and the Sentinel 2 from which shoreline positions were digitised from 01/01/1990 to 31/08/2019.

The speed and performance of the tool relies on the size of the observed area. The recommended area to obtain a sensible processing time is <100km^2^ (Fig 1). For this reason, the Krabi and NST coasts were divided into small cells of about 100 km^2^ as suggested by [19].

Collection dates of the shoreline positions varied dependant on area as a result of a range of factors. (1) The presence of clouds (only satellite images with less than 10% cloud cover were selected for digitisation). (2) The performance of the toolkit’s extraction, (tool is highly effective on straight, sandy beaches allowing the user to keep most of the shoreline digitised using the automated tool). However, because of the variety of environments along both coasts and the limits of the toolkit when the beach material changes from pre-sand, a careful visual inspection and filtering in mangrove areas was necessary to maintain the accuracy of the digitised shorelines. (3) The tidal level, in order to compensate for the lack of beach profiles that coastal managers would normally use to reposition the various digitised shorelines, it was decided to use information on the tidal level collected from various stations along the Krabi and the NST coasts in order to filter the digitised shoreline according to whether it was close to the high-water level mark or not. Hence, only digitised shorelines within a range of ±25 cm from the highest tides were kept. It is assumed that this method would allow comparisons between shoreline positions for conditions similar or very close to the highest tides. That range was also considered sufficient to obtain a good compromise between the number of shorelines kept and the spatial resolution of the methodology.

Shoreline change rates were then computed using the Digital Shoreline Analysis System (DSAS) extension of ArcMap [43]. The Linear Regression Rate in m/year (LRR) was selected from all the statistical parameters available within DSAS, as is commonly used for shoreline studies [59–65]. The LRR determines a rate-of-change statistic by fitting a least square regression to all shorelines at a specific transects. The LRR rates are determined by a best-fit regression line through the sample and have the advantages that: (1) it uses a minimum of three data points to produce any result [43], (2) all the data is used regardless of the trend and accuracy of the data, (3) it is very easy to understand and use [63]. Measurements were made at a regular spacing of 500 m along both coasts. It was decided that for the calculation of the rates of shoreline position change a minimum of four digitised shorelines were necessary to produce a representative result during the survey period. For this reason, very occasionally the spacing along the coasts between measurements was increased.

Despite the high performance of the tool one area (blue contoured area in Fig 1) in the Krabi province had to be digitised manually along a mangrove environment as attempts to automatically digitise multiple shorelines accurately across the survey timeline with the toolkit were not successful. This was predominantly related to the high degree of complexity in this area of the coastline (undulating).

**Fig 2:**
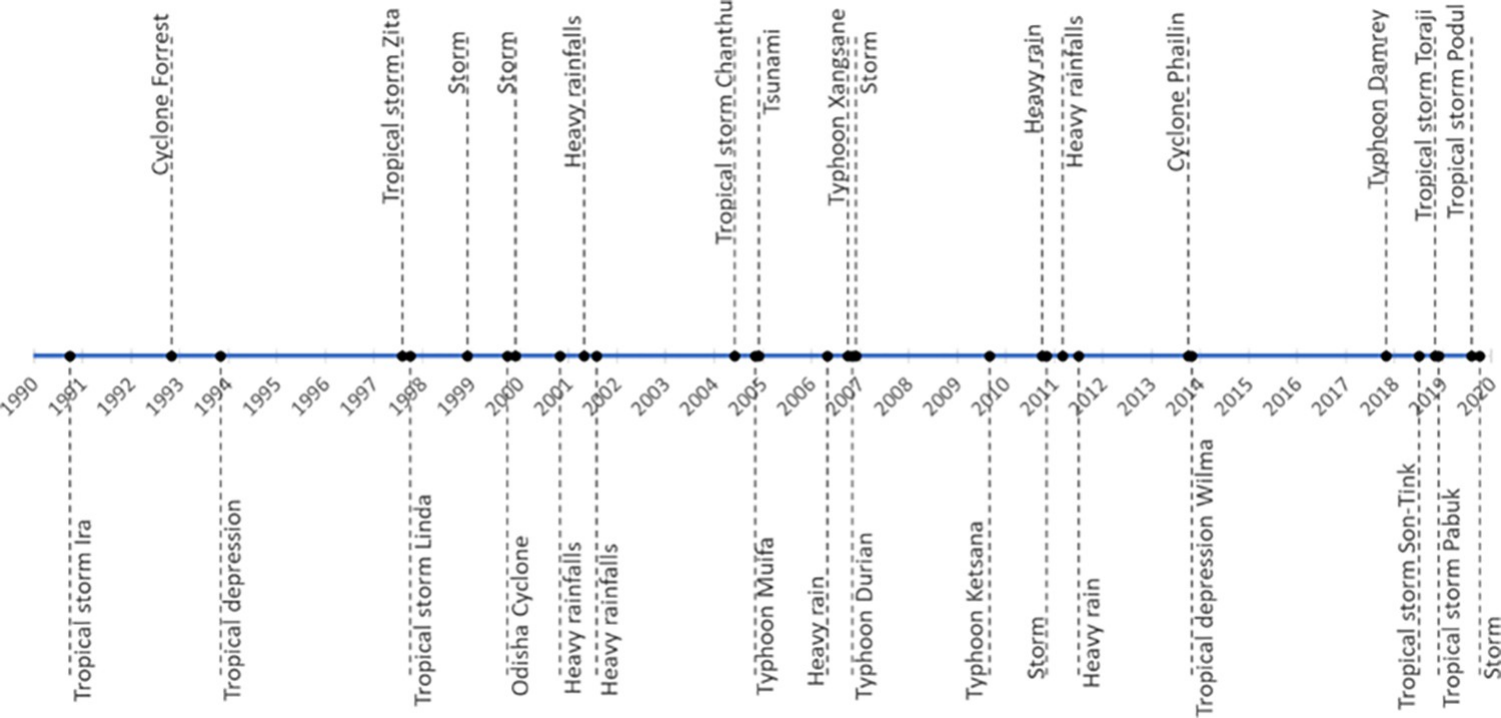
Extreme weather inventory. Data derived from on a conglomerate of news reports and the IBTrACTS data available on the National Oceanic and Atmospheric Administration (https://www.ncdc.noaa.gov/ibtracs/).

In order to link shoreline change to weather conditions or recorded natural disasters an inventory of extreme weather events was created including all land falling tropical storms and intense rainfall events that occurred during the survey period and the 2004 tsunami (Fig 2).

Due to the inherent inaccuracy of the sampling method used to digitise the shorelines by [19], it was decided to focus on larger shoreline changes recorded during the survey period (1990 to 2018) in order to match them to the most extreme weather events (Fig 2). The methodology uses Landsat 7 and 8 data with panchromatic image sharpening to improve the resolution (30 to 15 m). It also uses Landsat 5 (TM), which does not have a panchromatic band, so 30 m bands are down-sampled to 15 m by bilinear interpolation and finally, Sentinel 2 images that are down sampled by bilinear interpolation to 10 m. Only shoreline changes > 15 m, i.e. above the minimum the highest resolution, were used for matching with extreme weather and shoreline impacts.

## 4. Results

### 4.1 Spatial variations

#### 4.1.1 Nakhon Si Thammarat province

**Fig 3:**
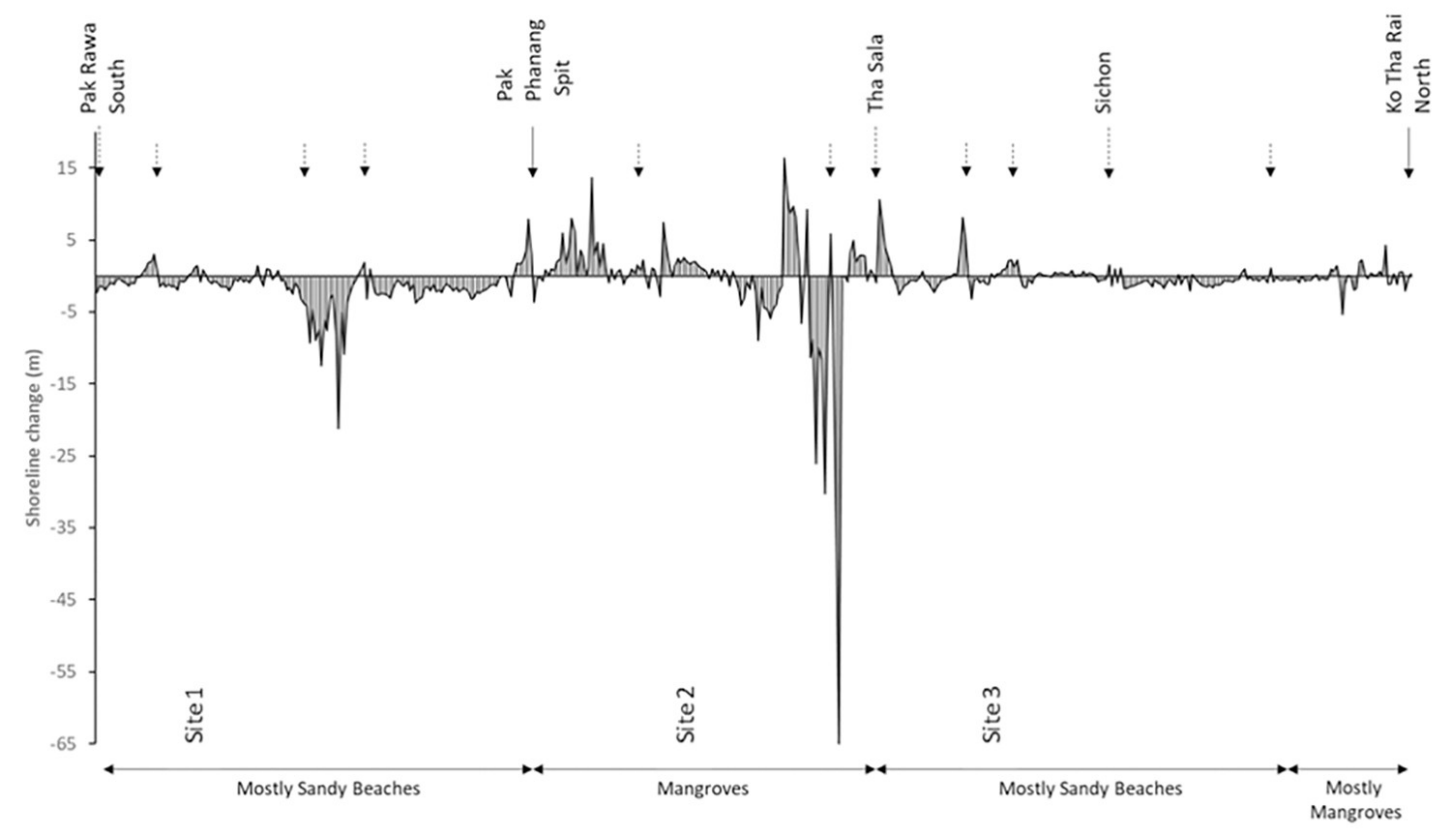
Shoreline change measured during the survey period along the Nakhon Si Thammarat coastline. The dashed arrows indicate the location of major river outlets. Plain arrows are used for geographic locations. The total length of coast is 236.8 km.

The coastline evolution model clearly displays a general longshore sediment transport going from South to North with erosion in the southern parts of the sub-sediment cells (updrift end) and progradation in the northern parts of these same cells (downdrift end, Fig 3).

In the South of NST (from Pak Rawa to Pak Phanang), the general trend along this stretch of sandy coast is erosion during the survey period. The beaches show erosion rates of up to -21.2 m/year while progradation rates go up to +7.9 m/year (Fig 3). Progradation areas are generally very localised in contrast to the long stretches of beach showing net erosion. These progradation areas are predominantly observed in proximity to features such as harbour arms, river mouths or coastal defence structures. The most noticeable progradation feature within this section of coast is the Pak Phanang spit. This spit expanded considerably in a NW direction between 2009 and 2018 by approximately 1 km, representing an expansion rate of approximately +111 m/year.

From Pak Phanang to Tha Sala, the coastline is mostly covered by mangroves (Fig 3) and erosion rates are the highest in this region (up to almost -66 m/year). As a general pattern, the inner part of the bay of Pak Phanang, from the Pak Phanang Spit to the Pak Nakhon river mouth, shows progradation with rates going up to 13.6 m/year. In contrast, the northern part of the bay, from the Pak Nakhon river mouth to Tha Sala, the shoreline changes show high rates of erosion.

The northern part of the Nakhon Si Thammarat coast (From Tha Sala to Ko Tha Rai), has a predominantly sandy beach coastline. However, erosion rates are much smaller than in the South of Nakhon Si Thammarat. Here, the coastline retreats at an average rate up to -3.2 m/year, while progradation downdrift of harbour arms or river mouths can be up to +10.6 m/year (Fig 3).

#### 4.1.2 Krabi province

**Fig 4:**
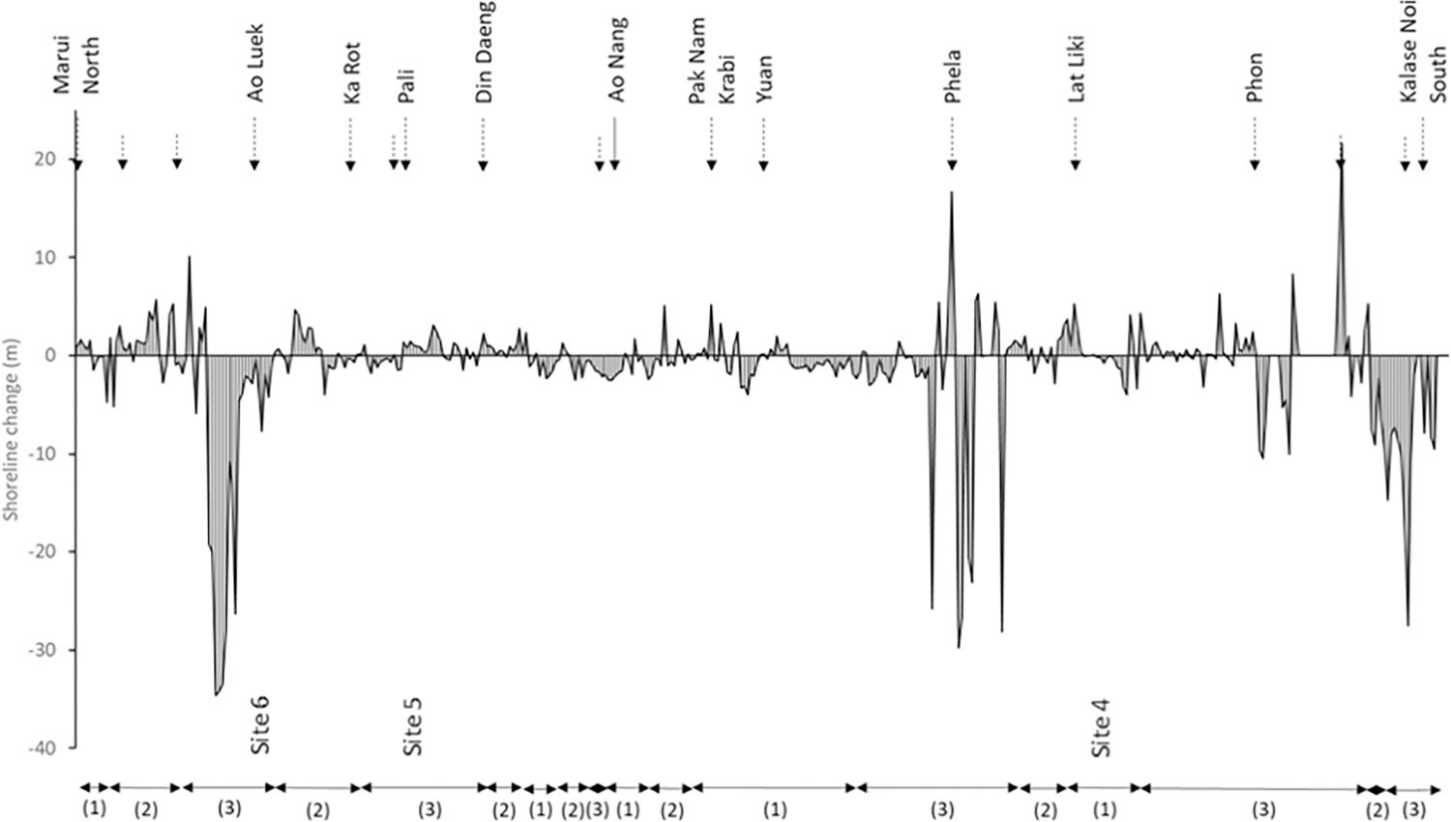
Shoreline change measured during the survey period along the mainland Krabi coastline. Dashed arrows indicate the location of major river outlets. Plain arrows are used for geographic locations. The total length of coast is 203.8 km. (1) shows a predominantly sandy beach coastline, (2) mostly rocky coast and (3) mostly mangrove coasts.

The coastline in the Krabi province is much more sinuous and diverse than Nakhon Si Thammarat. General trends suggest that mangroves in this area are much more dynamic than sandy beaches. Erosion and progradation rates in mangroves can go up to -34.5 and +21.7 m/year respectively. In comparison, the less dynamic pocket sandy beaches have lower erosion and progradation rates than those in NST, -4.1 and +4 m/year respectively (Fig 4).

### 4.2 Temporal variations

**Fig 5:**
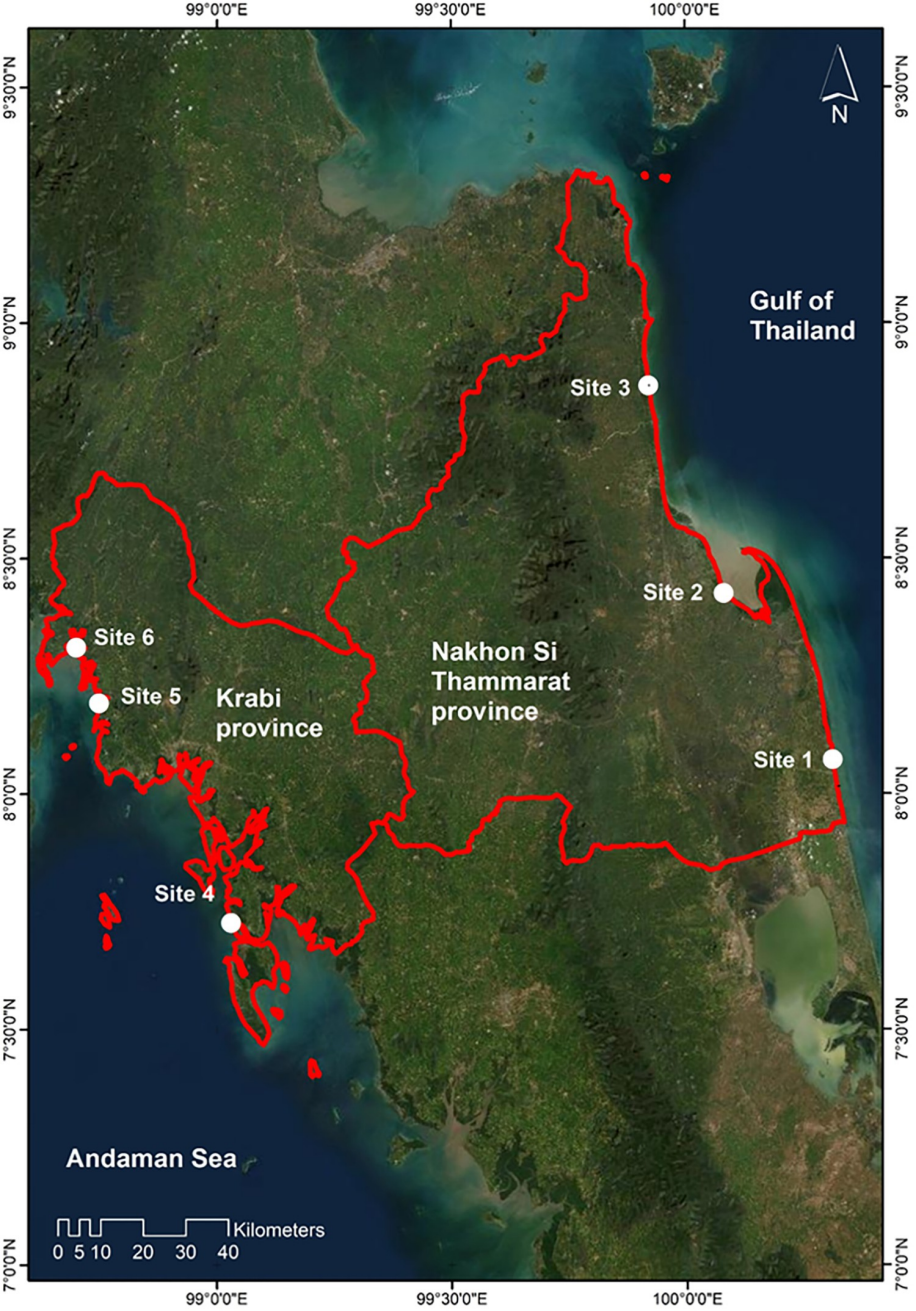
Sites selected for more detailed evaluation of processes influencing coastal dynamics. Basemap satellite images accessed from World Imagery ESRI Tile Layer. Credits: Esri, Maxar, GeoEye, Earthstar Geographics, CNES/Airbus DS, USDA, USGS, AeroGRID, IGN, and the GIS User Community.

In order to understand temporal changes and identify the different responses to weather or coastal defence management, it was decided to look at a variety of locations and environments along both coasts. Therefore, key sites were selected according to criteria such as the type of coastal environment i.e. mangrove or sandy beach, the presence or not of development or coastal defences, and finally whether the site was showing predominantly erosional or progradational trends over the survey period based on the results produced using Digital Shoreline Analysis System (DSAS, Fig 5). The aim was to evaluate different shoreline responses under varying environmental conditions but also to evaluate the reliability of the models produced based on the current understanding of sediment transport along the NST and Krabi coastlines.

#### 4.2.1 Nakhon Si Thammarat province

**Fig 6:**
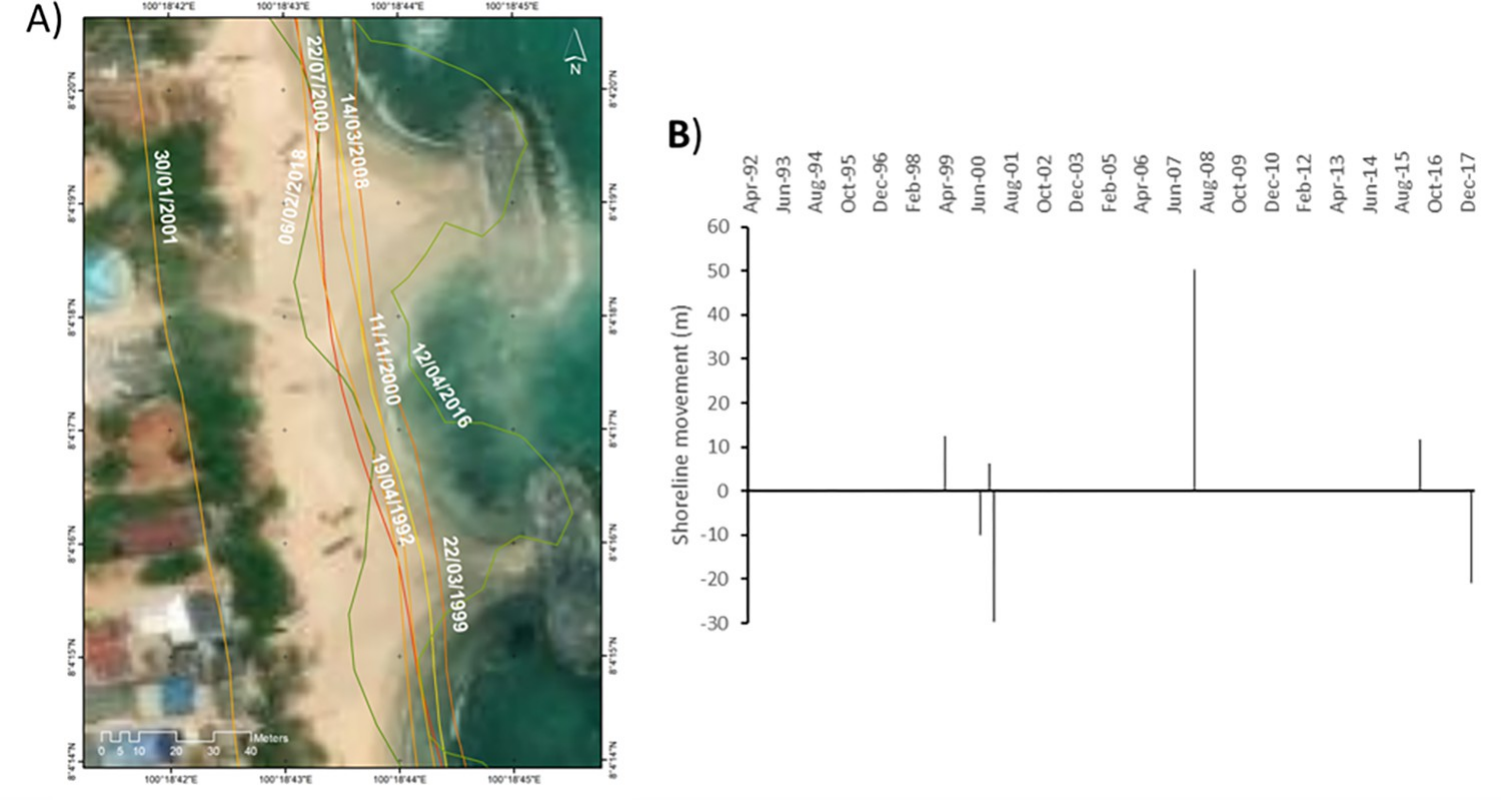
Representations of the shoreline movements observed during the survey period in site 1. A) Visualisation of the shoreline positions for the highest water level marks digitised with CoastSat. B) Shoreline movements measured using DSAS between two consecutives chronologically ordered digitised shorelines; negative values correspond to erosion and positive values to accretion. Basemap satellite images accessed from World Imagery ESRI Tile Layer. Credits: Esri, Maxar, GeoEye, Earthstar Geographics, CNES/Airbus DS, USDA, USGS, AeroGRID, IGN, and the GIS User Community.

**Site 1:** A Sandy beach in an area managed with hard engineered structures (Fig 6).

The digitised shorelines across the survey period show that from Apr 1992 to Mar 1999, the high-water level mark moved seaward by approximately +12.4 m, a trend that reversed by Jul 2000 showing a landward movement of the shoreline by -10.1 m. That erosional movement is synchronised with the occurrence of the Odisha cyclone in OCT-Nov 1999. Between Jul 2000 to Nov 2000, which corresponds to a SW monsoon season, the shoreline showed a seaward migration of approx. +6.4 m. Between Nov 2000 and Jan 2001, the shoreline retreated dramatically landward by -51.2 m. During this period, the monsoon of 21–25 Nov 2000 caused extremely heavy rain in the southern part of Thailand. It is more likely that these 4 days of extreme monsoon conditions affected dramatically the position of the shoreline. Between Jan 2001 and Mar 2008, the shoreline went almost back to its Nov 2000 position by moving seaward by +50.4 m. A variety of extreme events occurred during this period (Fig 2) and characterising the effect of these events over that long time period is difficult. However, it can be noticed that prior to Mar 2008, no extreme weather events were recorded for about one year, hence highlighting a potential time for beach volume recovery. Between Mar 2008 and Apr 2016, the shoreline moved seaward by +11.8 m in the space between the breakwaters and by +48 m directly in their proximity. Those breakwaters were built parallel to the shoreline helping the coastline to prograde despite the occurrence of a variety of extreme weather events such as typhoon Ketsana in 2009 (Fig 2). Between Apr 2016 and Feb 2018, the shoreline retreated landward by -20.9 m in the space directly in between two consecutive breakwaters and by -52 m directly behind the coastal defence structure. Typhoon Damrey was the dominant extreme weather event recorded during that period and this completely reversed the initial positive impact of the breakwater on the shoreline position.

**Fig 7:**
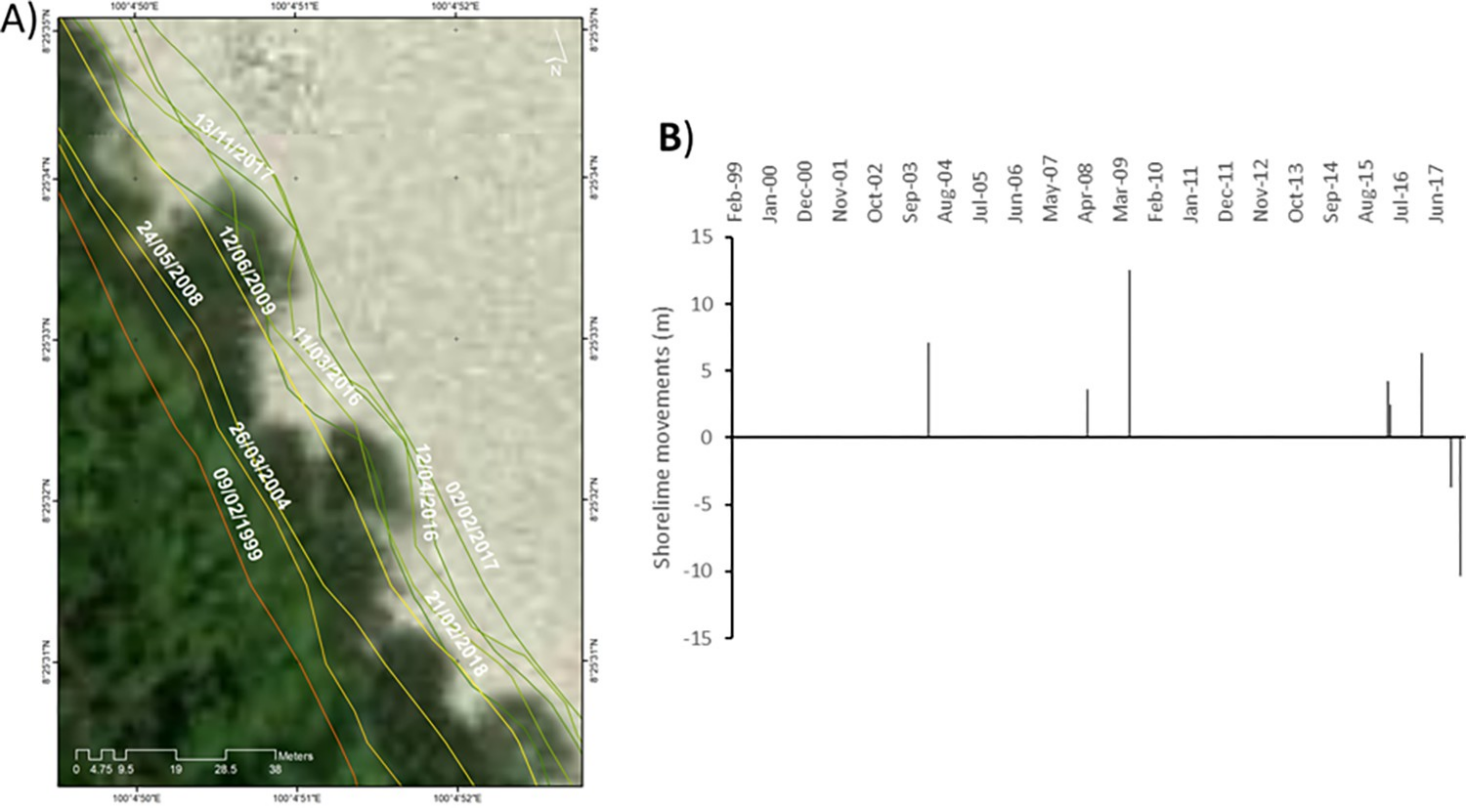
Representations of shoreline movements observed during the survey period in site 2. A) Visualisation of the shoreline positions for the highest water level marks digitised with CoastSat. B) Shoreline movements measured using DSAS between two consecutives chronologically ordered digitised shorelines; negative values correspond to erosion and positive values to accretion. Basemap satellite images accessed from World Imagery ESRI Tile Layer. Credits: Esri, Maxar, GeoEye, Earthstar Geographics, CNES/Airbus DS, USDA, USGS, AeroGRID, IGN, and the GIS User Community.

**Site 2**: A mangrove environment showing progradation (Fig 7).

This environment is located in Pak Phanang Bay, just north of several large river catchments, (Pak Phanang, the Cha Mao and the Bang Yai). The various positions of the shoreline reveal that between Feb 1999 and Mar 2004, the high-water level mark moved seaward by +7.1 m. During that period, no extreme weather affecting the East coast of Thailand was recorded. Between Mar 2004 and May 2008, the shoreline moved seaward by an additional +3.6 m, then +12.5m by Jun 2009, +4.2 m by Mar 2016, +2.5 m by Apr 2016 and +6.3 m by Feb 2017. During this period of time, a range of extreme weather events occurred along the coasts of Thailand (Fig 2) but clearly, they had minimal impact on the coastline. In contrast, between Feb 2017 and Nov 2017, the shoreline moved back landward by -3.7 m and then between Nov 2017 and Feb 2018 it moved again landward by an extra -10.4 m. Nov 2017 is mostly marked by the occurrence of the typhoon Damrey creating hydrodynamic conditions powerful enough to reach in the inside of the bay and erode some of the mangrove.

**Fig 8:**
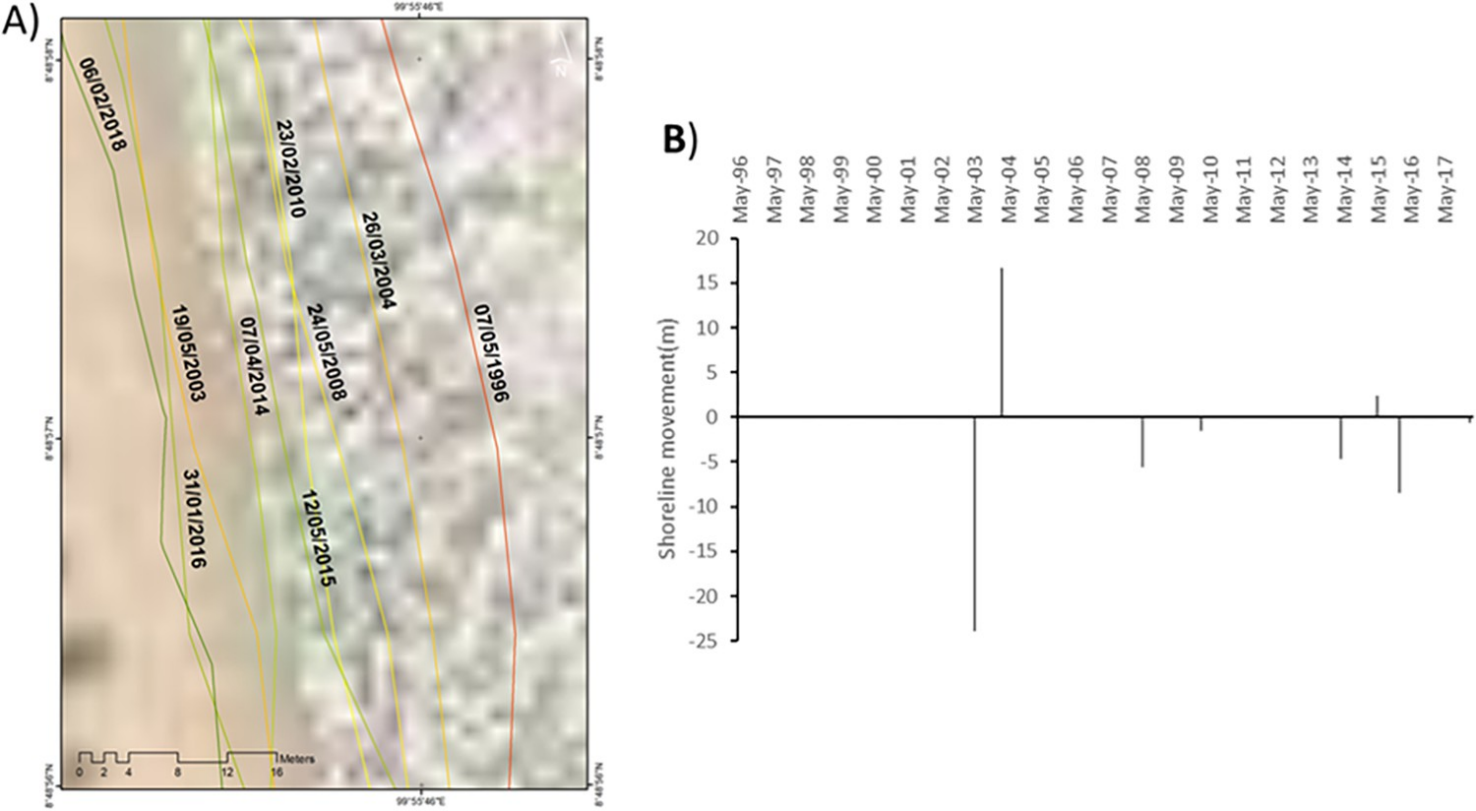
Representations of the shoreline movements observed during the survey period in site 3. A) Visualisation of the shoreline positions for the highest water level marks digitised with CoastSat. B) Shoreline movements measured using DSAS between two consecutives chronologically ordered digitised shorelines; negative values correspond to erosion and positive values to accretion. Basemap satellite images accessed from World Imagery ESRI Tile Layer. Credits: Esri, Maxar, GeoEye, Earthstar Geographics, CNES/Airbus DS, USDA, USGS, AeroGRID, IGN, and the GIS User Community.

**Site 3**: A natural sandy beach showing net erosion (Fig 8).

This site is located in the sedimentary sub-cell just updrift of the Hin river; more explicitly it is about 5 km South from the Hin river mouth and exhibits net erosion between May 1996 and Feb 2018 measured at -2.3 m/y. From May 1996 to May 2003, the shoreline moved landward by approximately -24 m. During this time period three main extreme weather events occurred in Thailand, tropical storm Linda in Aug 1997, tropical storm Zita in Oct 1997 and the Odisha cyclone in Oct 1999. Between May 2003 and Mar 2004, the shoreline moved seaward by +16.7 m. During this period no extreme events were recorded. Between Mar 2004 and Apr 2014, the shoreline kept moving landward at -5.6 m in May 2008, -1.6 m in Feb 2010 and -4.7 m in Apr 2014. During this period, the site was impacted by typhoon Muifa in Nov 2004, tropical storm Chanthu in Jun 2004, an extremely rainy monsoon in May 2006, typhoon Xangsane in Oct 2006, typhoon Durian in Nov 2006, followed by more stormy weather in Dec 2006, typhoon Ketsana in Sep 2009, a heavy rainfall event in Oct 2010 followed by storms in Dec 2010, more heavy rains in Mar and Jul 2011, cyclone Phailin in Oct 2013 and finally tropical depression Wilma in Nov 2013. It seems highly probable that those events contributed to creating conditions that progressively eroded that section of coast. Between Apr 2014 and May 2015, the shoreline moved seaward by +2.4 m and again in that time of accretion, no noticeable extreme weather events were recorded. The shoreline moved landward by -8.4 m until Jan 2016 and a further -0.6 m in Feb 2018. No noticeable extreme weather event was recorded between May 2015 and Jan 2016 but afterwards, between Jan 2016 and Feb 2018, Typhoon Damrey was recorded in Nov 2017 which may have been responsible for the erosion observed at that time.

#### 4.2.2 Krabi province

**Fig 9:**
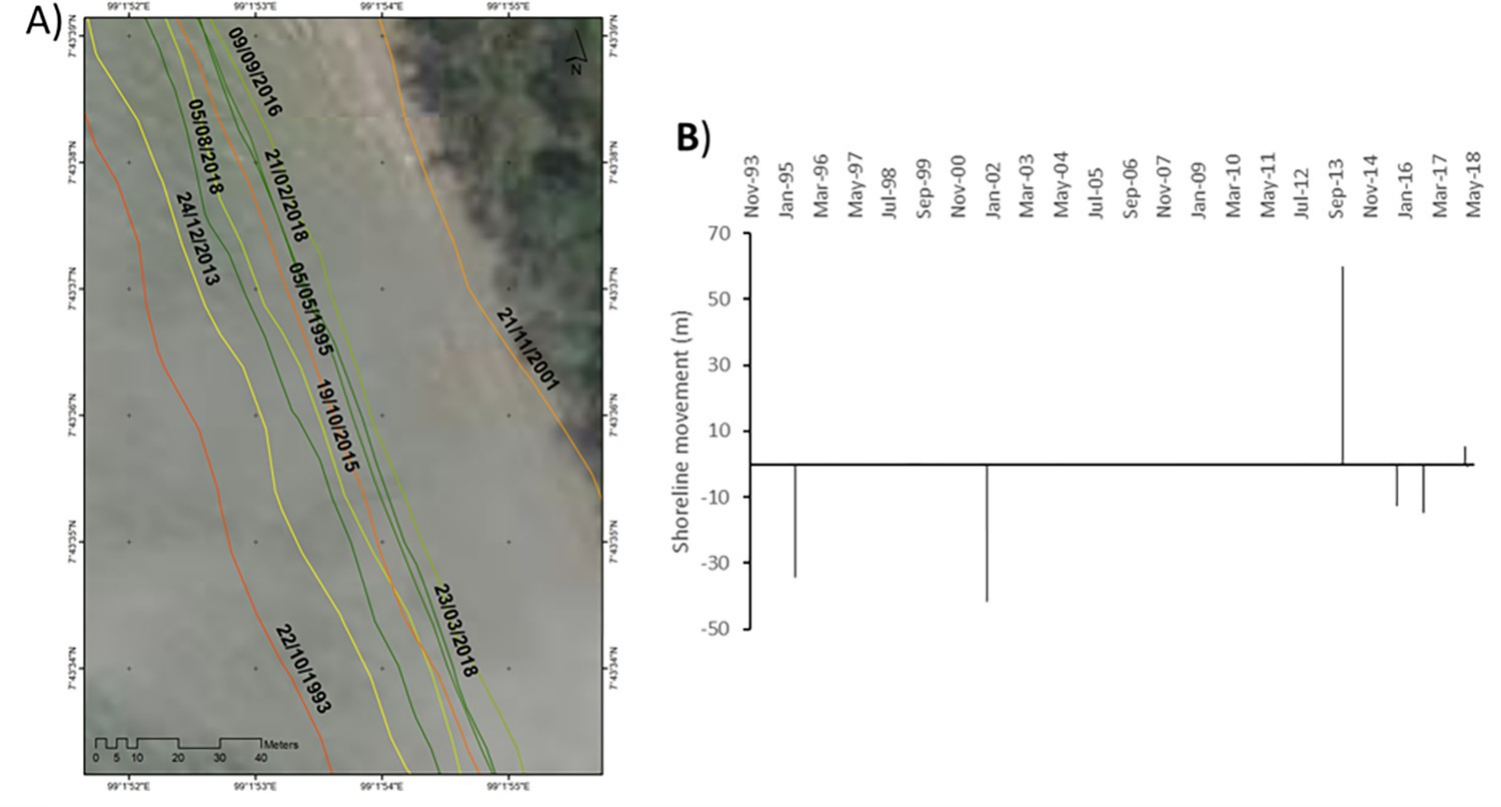
Representations of the shoreline movements observed during the survey period in site 4. A) Visualisation of the shoreline positions for the highest water level marks digitised with CoastSat. B) Shoreline movements measured using DSAS between two consecutives chronologically ordered digitised shorelines; negative values correspond to erosion and positive values to accretion. Basemap satellite images accessed from World Imagery ESRI Tile Layer. Credits: Esri, Maxar, GeoEye, Earthstar Geographics, CNES/Airbus DS, USDA, USGS, AeroGRID, IGN, and the GIS User Community.

**Site 4**: A natural sandy beach with no hard engineering (Fig 9).

This site corresponds to an open sandy beach just north of Ko Lanta. This site displayed very little change (-0.2m/year) over the whole study period (Oct 1993 to Aug 2018). However, when looking in detail, some clear shoreline changes can be highlighted. Between Oct 1993 to May 1995, the shoreline moved landward by approximately -34.5 m. This period is marked by the occurrence of a tropical depression in Nov 1993 that is likely the cause of the erosion. Between May 1995 and Nov 2001, it moved landward by an extra -41.9 m. This time period is marked by the occurrence of tropical storm Zita in Aug 1997, tropical storm Linda in Oct 1997, the Odisha cyclone in Oct 1999 followed by strong storms in Dec 1999 and finally more extreme monsoon weather in Nov 2000, May 2001 and Aug 2001. Between Nov 2001 and Dec 2013, several extreme weather events occurred including, tropical storm Chanthu in Jun 2004, typhoon Muifa in Nov 2004, extreme monsoon weather in May 2006, typhoon Xangsane in Oct 2006, typhoon Durian in Nov 2006 followed by extreme stormy weather in Dec 2006, typhoon Ketsana in Sep 2009, more extreme monsoon weather in Oct/Nov 2010, Mar 2011 and Jul 2011 and finally just before the end of that time period cyclone Phailin in Oct 2013. At the end of that time period the shoreline moved seaward by +60 m. From Dec 2013 to Sep 2016, the shoreline moved landward -12.9 m by Oct 2015 and another -14.8 m by Sep 2016. During that time period, no obvious extreme weather event was recorded. From Sep 2016 to Feb 2018, a seaward movement of the shoreline position is measured at +5.5 m while typhoon Damrey passed Thailand in Nov 2017. Finally, between Feb 2018 and Aug 2018, the shoreline moved seaward by an extra +13 m while the tropical storm Son-Tink passed Thailand in Jul 2018.

**Fig 10:**
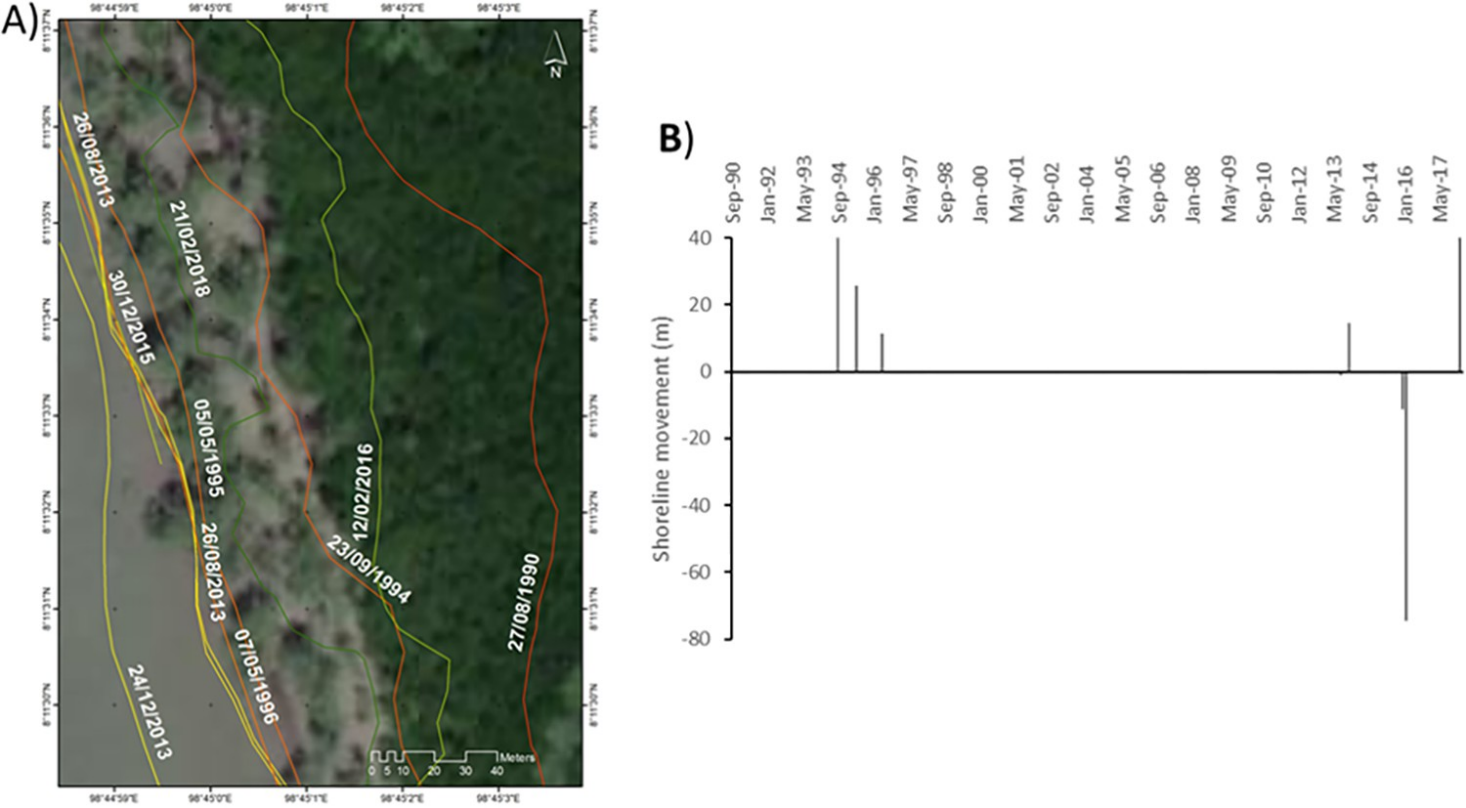
Representations of the shoreline movements observed during the survey period in site 5. A) Visualisation of the shoreline positions for the highest water level marks digitised with CoastSat. B) Shoreline movements measured using DSAS between two consecutives chronologically ordered digitised shorelines; negative values correspond to erosion and positive values to accretion. Basemap satellite images accessed from World Imagery ESRI Tile Layer. Credits: Esri, Maxar, GeoEye, Earthstar Geographics, CNES/Airbus DS, USDA, USGS, AeroGRID, IGN, and the GIS User Community.

**Site 5**: Mangrove environment showing overall progradation (Fig 10).

This site is located in the Bay of Phang Nga just South of the Pali mangrove channel. Over the whole survey period, from 1990 to 2018, the mangrove prograded by approximately +1.5 m/year. When looking at the details of the shoreline positions during this time period, some strong movements of the shoreline position were identified. Between Aug 1990 and Sep 1994, the shoreline moved seaward by +92.5 m. During that time, tropical storm Ira in Oct 1990, cyclone Forrest in Nov 1992 and a tropical depression in Nov 1993 travelled through Thailand. By May 1995, the shoreline moved further seaward by +25.6 m and another +11.4 m by Mar 1996. During that time period, no extreme weather was recorded. Between May 1996 and Aug 2013, a small landward movement was measured at -1.1 m but that trend reversed between Aug 2013 and Dec 2013 with a seaward movement of +14.5 m. Between May 1996 and Aug 2013, a variety of extreme weather events were recorded (Fig 2). The time period corresponding to the reversal of shoreline movement (Aug to Dec 2013) matches with the occurrence of cyclone Phailin and the tropical depression Wilma. Between Dec 2013 to Feb 2016, the shoreline moved significantly landward with movements measured at -11.4 m by Dec 2015 and another -74.5 m thereafter. These changes do not seem to be driven by any extreme weather conditions. Between Feb 2016 and Feb 2018, the shoreline position moved seaward again by +39.9 m and the only extreme weather inventoried for that time period is typhoon Damrey in Nov 2017.

**Fig 11:**
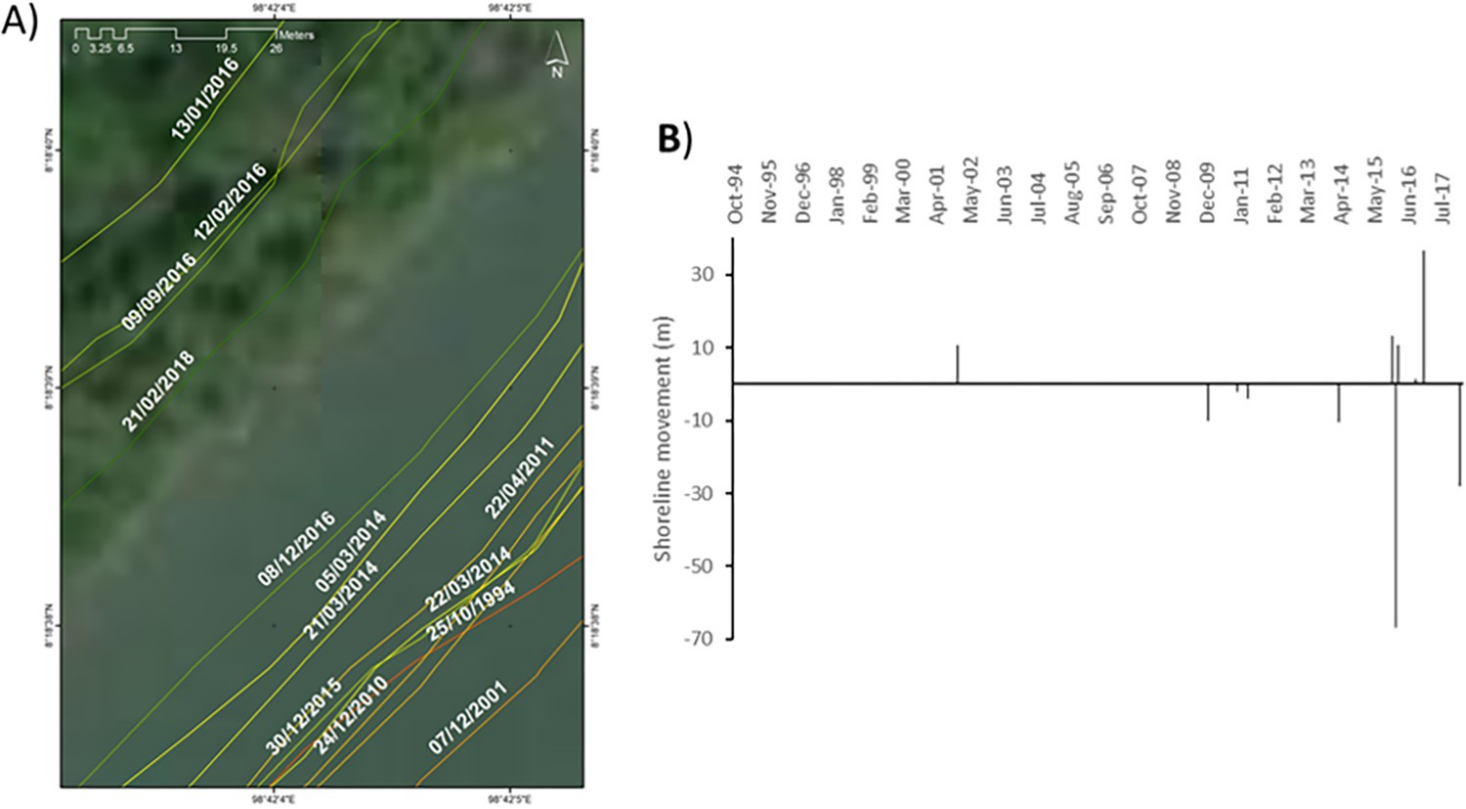
Representations of the shoreline movements observed during the survey period in site 6. A) Visualisation of the shoreline positions for the highest water level marks digitised with CoastSat. B) Shoreline movements measured using DSAS between two consecutives chronologically ordered digitised shorelines; negative values correspond to erosion and positive values to accretion. Basemap satellite images accessed from World Imagery ESRI Tile Layer. Credits: Esri, Maxar, GeoEye, Earthstar Geographics, CNES/Airbus DS, USDA, USGS, AeroGRID, IGN, and the GIS User Community.

**Site 6**: Mangrove environment showing overall erosion (Fig 11).

This site is a mangrove environment that presents net erosion over the study period (LRR approx. -2.4 m/year). Between Oct 1994 and Dec 2001, the shoreline moved seaward by +10.7 m. This rather long time period was marked by a range of extreme weather events (Fig 2). Between Dec 2001 and Mar 2014, the shoreline moved landward by -10.3 m until Jan 2010, and a further -2 m by Dec 2010, -4 m by Apr 2011 and finally another -10.4 m by Mar 2014. The chronology of the weather events (Fig 2) show that the years 2004, 2006, 2009, 2010, 2011 and 2013 are marked by extreme events. A seaward movement is once again observed between Mar 2014 and Dec 2015 measured at +13.3 m only to reverse significantly in Jan 2016 with a retreat landward measured at -66.8 m. From Jan 2016 to Dec 2016, the shoreline continued moving seawards by a total of +49 m. During the time period between Mar 2014 and Dec 2016, no extreme weather events were recorded. Finally, between Dec 2016 and Feb 2018, time during which typhoon Damrey struck the coast, the shoreline moved landward by -28.1 m.

## 5 Discussion

### 5.1 Performance of the tool

CoastSat [19] is an open source software toolkit enabling the digitisation of a time series of shoreline positions on sandy beaches using open access satellite imagery within the Google Earth Engine platform. The use of the toolkit along the coasts of NST and the Krabi province proved to be highly successful with the digitisation of a large number of shoreline positions across a range of environments for a period of nearly 30 years. Shoreline positions digitised in mangrove environments were very carefully checked as the toolkit was only designed for sandy beaches. This careful review resulted in discarding a large number of shorelines or sections of shoreline in mangrove environments. However, this still provided enough accurately digitised shorelines along those environments to be used in the Digital Shoreline Analysis System (DSAS) software to deliver a comprehensive time series for shoreline change.

The precision of the digitised shorelines is down to a sub-pixel resolution of approx. 10 m [19]. Therefore, this tool is designed to highlight significant shoreline change on extremely dynamic coastlines during short time periods or lower magnitude shoreline change trends over longer time series. Both the Krabi and Nakhon Si Thammarat coasts have been reported by local authorities (DMCR) to be suffering from severe erosion, for example it has been reported that average rates of erosion were > 5 m for the years 2011 and 2017 along the NST coast. The results of this study agree and confirm those rates and locations of erosion albeit over a greater time period and spatial scale.

### 5.2 Spatial variations

#### 5.2.1 Nakhon Si Thammarat

Progradation was found to be occurring at the downdrift end of coastal structures or river mouths as a direct consequence of South to North longshore sediment transport. The stretch of coastline between the Pak Rawa river to the Pak Phanang Spit is undergoing noticeable erosion highlighting a discrepancy between removal and supply of sediment within the sediment cell (Fig 3). Potential sources of sediment are from marine origin, i.e. the Gulf of Thailand, and fluvial discharge in the NST and Songkhla provinces. Unfortunately, no quantitative information was found on sediment transport to the shore from marine origins along the NST coast; however, it is suspected that storms and extreme weather from the China Sea bring in sediment from the Gulf of Thailand towards the shore due to the shallow bathymetry and the prevailing currents. A rapid inspection of the coastline and the presence of effluents shows that no major or noticeable terrestrial outlets would provide enough sediment to counterbalance the effect of longshore sediment transport, which is likely to be a factor in the considerable rates of erosion observed along this coast.

Another parameter to consider is human activity along the NST coast. This coastline is subject to intensive shrimp farming activities and [27] identified that these contribute to the rates of erosion observed during the survey period (“50 m erosion in one day”) as when their seaward border is destroyed, the sea invades these ponds. In other regions it has been noted that environmental pollution related to shrimp farms has a degradational effect on coastal vegetation and this can result in loss of mangrove cover and erosion [66]. [27] highlighted that illegal excavations of sand along the coast for commercial purposes was a regular occurrence, as has been noted for other areas in SE Asia [33]. These activities could in part explain sudden or large erosion events in this area. Finally, the poor design or placement of coastal defence structures along the coast have not achieved the desired outcomes [27]. When looking at the effect of coastal defence structures along the NST coast, three main types of structure can be seen: seawalls, breakwater walls and groynes/harbour arms. Changes in shoreline positions during our survey period revealed that breakwater walls initially slowed down coastal erosion in their vicinity, although the shoreline quickly continued to move landward within a year or two (Site 1).

The most noticeable progradational feature in the South of NST is the spit of the Pak Phanang bay. Its consistent growth has allowed the deposition of fine sediment inside the bay and protecting the land directly behind from high wave energy influences. Mangrove forests and their degradation and loss have been of a great concern for government authorities in Thailand since the 1990’s. Mangroves are very important for Thai communities as they provide important cultural, financial, tourism and ecological resources, not to mention their key role in carbon sequestration [37]. [67] and then [68] reported that more than 50% of the mangrove forest cover in Thailand was lost between 1961 and 1993. Recognising the urgency to protect, restore and better manage those environments, in 1996 Thailand introduced new environmental policies and invested a large amount of time, research, money and manpower to restore mangroves in the country. However, mangrove forests are very hard to re-establish being sensitive to variations in environmental parameters, such as sedimentation rates, water quality, water levels, human activities etc. and where stressors still occur, restoration practices are unlikely to be successful. [69] reported annual losses of mangrove forest areas as 0.71% in Thailand, in spite of government led protection and restoration programmes. Our results evaluate rates over a more recent time period, from 1990 to 2018. The mangrove forest inside the bay of Pak Phanang is developing slowly (Fig 3) with only very localised areas displaying erosion. These predominant progradational trends are likely to be the result of combined actions taken by the authorities, conservation policies, public awareness and better management such as restoration by planting new seedlings, showing a potential local reversal of the loss and degradation trend of mangroves in Thailand.

Just North of the bay, the very high erosion rates recorded in this study are related to the linking of back barrier lagoons within the mangrove to the sea as a result of wave activity (Fig 3).

The Northern part of the NST coastline from Tha Sala to Sichon clearly shows erosional trends in the updrift end of sub-sedimentary sediment cells and accretive trends in the downdrift end, typically in accordance with the residual longshore sediment transport along that coast. Rates of progradation and erosion are fairly balanced, both being between 0 and 5 m/year. in contrast to the Southern part of NST, this length of coast is dissected by a greater number of significant river outlets bringing material to the coastal sedimentary sub-cells. From Sichon to Tha Rai, erosion is a recurrent feature, clearly highlighting sediment starvation in those sub-cells. Most beaches in that section of the coast are pocket beaches sitting between rocky limestone substrate. As such, the sediment supply is very limited from terrestrial sources with only two river outlets, the Sichon and the Khun Nom.

As an overall observation, the NST coast is severely impacted by erosion and despite clear attempts to slow down this process by building up coastal defence structures and beach nourishment, rates of erosion are still within the same order of magnitude as those recorded in the past [27].

#### 5.2.2 Krabi

The Krabi coastline has quite a different setting than NST. Aside from the obvious differences in hydrodynamics, sediment supply, coastal geology and geomorphology, mangrove forests are more prevalent. The evolution of the shoreline noted from this study mostly highlights erosion in accordance with previous earlier studies [26, 67, 69]. As mentioned earlier, mangrove forests in Thailand have largely been reported as decreasing in extent as a result of anthropic pressures such as shrimp farming, tin-mining, palm plantations, paddy fields, urbanisation, reduced riverine inputs and natural processes wave activity and prevailing monsoon. All these activities are common in the Krabi province and have certainly affected the sustainability of the mangrove forests along the coast, and tin mining has been reported as a major reason for mangrove degradation [70]. [71] produced an inventory of the surface area covered by mangroves from 1961 to 2007 by region in Thailand. Their results showed that in Krabi, mangrove areas decreased by about 30% between 1961 to 1996, but expanded by 26% between 1996 and 2007. Our results represent an extension in the timeline of this work, however the overall trend measured by our study from 1990 to 2018 is still erosion, not progradation, albeit fairly low (predominantly between 0 and -5 m). The highest rates of erosion (i.e. >-10 m/year) seem to be more common in or very near estuaries. A lot of the Krabi economy revolves around water activities and estuarine channels are heavy traffic navigation ways in that coastal area. As such, dredging activities are a very common and regular practice as well as wake swash waves, which may provide an explanation for the significant erosion rates around estuaries. It is important to note that when looking at the distribution of the areas of mangroves prograding vs areas of mangroves eroding, again it is clear that most mangroves along the Krabi coastline are receding but one rather significant area, between the Pali estuary and the Din Daeng estuary, displays predominant progradation (Fig 4). It is worth pointing out that the orientation of that section of coast may be an explanation of this mangrove resilience. Being aligned in a NNE direction, this section of mangrove coastline is sheltered from direct incoming wave incidence. When expanding this observation to the beach environments along the Krabi coastline it can be seen that most sections of this coastline that are aligned in a general NE direction seem to show trends of accretion too, which reinforce the suggestion that those areas are protected from most wave activity.

Despite the fact that both coasts display different geological, geomorphological and hydrodynamic environments as mentioned earlier, it is interesting to note that both coasts are, on average erosional. The principal differences are that the East coast (NST) is much more dynamic than the West coast (Krabi) for both erosion and progradation, this observation is in accordance to previous observations by [26]. This can be explained by the fact that despite having an average wave height lower than in the Andaman Sea, a greater number of strong storms, typhoons and cyclones make landfall on the NST coastline. The bathymetry and the geomorphology of the Western coast better protects the beaches of Krabi. The difference in erosion may also be linked to issues of coastal land subsidence along the Gulf of Thailand accentuating the erosional trend of the NST beaches.

### 5.3 Temporal variations

#### 5.3.1 Nakhon Si Thammarat

Unmanaged sites (site 3) clearly show the same trend, erosion is associated with the occurrence of extreme weather events such as heavy rain, typhoons, tropical storms, etc. At these sites, the greatest progradation occurs during periods when extreme weather events don’t occur. This underlines the effect of extreme weather events on the evolution of the shoreline along the NST coast.

Managed beaches such as site 1, despite the presence of coastal defence, clearly highlight the short-term erosive impact of typhoons on the coastline. This highlights that the effectiveness of the coastal defence structures in place is weak against such weather conditions. The overall evolution of the shoreline in this area also highlights that these coastal defence structures have not allowed to beach to reach a stable position during the period of our survey, which leaves doubts concerning the long-term effectiveness of this management method at this site. These observations confirm those of [27], highlighting the difficulties of designing effective solutions for coastal erosion in Thailand because of a variety of pressures from funding, stakeholder’s welfare, governance effectiveness, and the lack of expertise or working models.

Our results from the inside of Pak Phanang bay (Site 2) support observations previously noted by [72], describing a progressive recovery of the mangrove forests between 1995 and 2004. That progression was despite illegal dumping of shrimp farm waste in the bay in 1996 that led to considerable damage to the mangrove forest [73]. However, the combination of both, mangrove restoration and sediment supply from the rivers resulted in a decrease in the open water area of the bay [73] and that progradation continued until 2017. Between 2017 and 2018, our results suggest that the shoreline moved back landward as a result of erosion of the mudflats. It is not clear what the cause is, however it is important to acknowledge this erosional movement is under the sub-pixel size predetermined earlier and therefore that movement could simply be related to the error margin of the methodology.

#### 5.3.2 Krabi

The impact of storms or extreme weather events along this coast was much more difficult to understand. Both erosional and progradational movements seems to be occurring irrespective of the occurrence extreme weather conditions. The clear relationship between extreme weather and erosion in areas with no hard-engineered defence structures observed in NST is not as clear along the Krabi coast. The Krabi coast as mentioned earlier is much more complex than the NST coastline. The sinuosity of the coastline, the shallowness of the Andaman sea, the presence of multiple islands directly in front of the Krabi coastline, the geomorphology, the complexity of the currents, the tidal regime, the generally higher wave energy delivered to the coast, the monsoons and finally human activities create a complex and unique hydrodynamic and sedimentary system. Generally, extreme wave energy is largely dissipated by the gentle coastal shelf slope and shallow bathymetry of the Andaman Sea in comparison to the Gulf of Thailand. The various islands in the Andaman sea and the Phuket peninsula also contribute to the protection of the Krabi coastline during extreme weather events. Both effects make the relationship between storm and shoreline change less evident than on the NST coast. Current flows are generally directed North-Westward towards the Andaman Sea near the Krabi coastline, which transport sediment in a Northward direction during calm or stormy wave conditions; but again, sediment accumulation at the downdrift end of sub-sediment cells or the rare coastal structures is not conclusive along the Krabi coastline, most likely due to its sinuosity. However, it seems to be clear that the lengths of the coastline broadly facing a NW direction are more protected from waves and currents in this area as they generally show net accretion.

The mangroves (Sites 5 and 6) were affected by significant erosion between Dec 2015 and Jan 2016 (respectively -74.5 and -66.8 m). That period does not relate to any extreme weather events when this study investigated weather records in Krabi, suggesting that the sudden change in shoreline position was linked to other factors. The coastline of Krabi province has been under a lot of pressure from human activity. Many of these pressures, such as dredging, shrimp farming, mining or dam building, influence sediment supply and distribution and/or mangrove condition. It is possible that human activities are the cause of these shoreline retreats but no conclusive proof of the root of the impacts has been found by this study.

## 6 Conclusions

CoastSat is an effective and robust toolkit to monitor shorelines, especially when the survey area is very large and manual digitisation would take too long to achieve. Automatized techniques also allow to mitigate the inherent errors associated with manual digitisation. Using this tool, this study was able to digitise approx. 560 km of shoreline positions for every sufficiently cloudless free satellite image available (<10%) over a period of 29 years. In the context of COVID 19 travel bans, and lockdowns, this toolkit permits remote quantitative and qualitative measurements of shoreline changes without the necessity to travel and directly assess shoreline dynamics.

The combination of CoastSat and Digital Shoreline Analysis System (DSAS) permitted an update and confirmed trends of coastal erosion that have concerned local and national authorities and stakeholders since the 1980s. Rates of erosion are significant, especially along the sandy beaches of NST, threatening local coastal communities in this province. In Krabi, that threat is similar with a net retreat of the coastline, despite the lower rates of erosion. Previous studies and reports of shoreline position change over shorter time periods are of a similar order as those obtained using CoastSat in our study, which is an indication of the precision of the toolkit. All these results show that the threat to coastlines in southern Thailand is high, and this is likely to be exacerbated by climate change and sea level rise as well as the issue of coastal land subsidence along the Gulf of Thailand.

Temporal variations in the shoreline reveal how sensitive shoreline evolution in NST is to extreme weather events coming from the China sea. It also highlights that the Krabi coastline is less affected by systematic erosion during similar events.

In the same spirit as [19] CoastSat toolkit, new automated tools are emerging, based on different algorithms and functionalities. CASSIE [20] for example is an online platform allowing the digitisation and computation of shoreline changes on any coast using Sentinel and LandSat free images around the world all in one package. Each tool has pros and cons that will decide which tool is more appropriate to individual research aims or preferences, but in a situation where travel is limited or access is complex and the availability of free remotely sensed data is increasing, these types of tools are becoming more and more invaluable for environmental research.
